# Generation of Abundant Defects in Ferrite Carbon Magnetic Nanomaterials for Eliminating Electromagnetic Interference in Communication

**DOI:** 10.3390/ma15196650

**Published:** 2022-09-25

**Authors:** Peng Yang, Qian Hao, Junsheng Zhang, Fang Liang, Xiaoning Bo, Peifen Wang

**Affiliations:** 1 Department of Electrical Engineering, Taiyuan Institute of Technology, Taiyuan 030008, China; 2 School of Information and Communication Engineering, North University of China, Taiyuan 030051, China; 3 Department of Electronics, Xinzhou Teachers University, Xinzhou 034000, China; 4 College of Environmental Science and Engineering, Taiyuan University of Technology, Taiyuan 030002, China

**Keywords:** ferrite magnetic nanomaterials, lightweight, controllable structure, defects, microwave absorbing properties

## Abstract

A series of novel ferrite carbon nanomaterials are considered to obtain the potential advantages in elimination of the electromagnetic interference effects. Herein, the iron nanoparticles coated on amorphous carbon were prepared by facile agar-gel synthesis. Meanwhile, the synergy between carbon supporting and ferrite nanomaterials could be proved to promote the absorption properties. Among all samples, the iron nanoparticles coated on amorphous carbon show the highest microwave absorption properties, achieving the maximum reflection loss (RL) of −14.3 dB at 6 GHz (5.5-milimeter thickness), and the bandwidths over −10 dB (90% absorption) was 2.5 GHz. Combining analysis results, it is confirmed that the as-prepared iron nanoparticles have the highest surface area, homogeneous distribution, abundant defect, and well-defined pore structure, which could significantly affect the absorption properties at 6 GHz. Furthermore, the abundant defects derived from the interface were the essential reason for the improved absorption properties. Overall, it provided a new strategy to design an effective method to absorb nanomaterials for the elimination of electromagnetic interference, especially the coordination of metal species and carbon supporting.

## 1. Introduction

With the wide application of electronic equipment and communication technology in various fields of life, the electromagnetic interference (EMI) is becoming more and more prominent, which not only seriously interferes with the stable work of precision equipment, but also dramatically endangers human safety [1,2,3,4]. To date, the preparation of high-performance microwave absorption material is the most effective strategy to eliminate the electromagnetic interference problems, which could protect the electromagnetic fields generated by electronic devices, thereby achieving the blocking of electromagnetic waves and/or energy attenuation [5,6,7]. In recent years, microwave absorption materials have become a critical research topic and continuable development direction due to its light weight, thin thickness, high-efficiency absorption intensity, and wide bandwidth.

Recently, numerous research of various microwave absorption materials mostly focuses on ferrite absorbing materials [8,9,10,11], metal magnetic absorbing materials [12,13,14,15], conductive polymer absorbing materials [16,17], carbon-based absorbing materials [18], and other lightweight wideband absorbing materials [19,20]. Among them, the kinds of ferrite materials have attracted widespread attention because it reveals the efficient activity for microwave absorbing; nevertheless, its application is limited by weight. Therefore, decreasing the weight and improving the dispersity of ferrites based on material could be considered to promote the microwave absorption performance. In addition, the properties of ferrite materials can be simulated by machine learning and subsequently verified by experiments to facilitate the prediction of absorbing properties [21,22,23]. Meanwhile, carbon-based absorbing materials have been regarded as the best optimal for EMI shielding due to their advantages such as light weight, outstanding flexibility, specific surface area, economy, and facile preparation. Herein, the available large surface and light weight of carbon-based materials have an advantage to combine with ferrites for high-performance wave absorbing absorbents, which will improve the width of the absorption band [24,25,26,27]. Therefore, it is imperative to exploit the mixture absorbents of ferrite and carbon for light weight with a well-defined and controllable structure.

Many traditional strategies have been proposed for the preparation of ferrite magnetic nanoparticles, such as coprecipitation method [28,29,30], sol-gel method [31,32,33], hydrothermal method [34], and solvothermal method [35,36], to improve the absorbing property of various mixed metal oxides. Inspired by the above method, an agar-gel synthesis approach was developed to facilitate the mixture of ferrite and carbon nanomaterials. We explored the influence of different metal species on morphology, phase structure, and surface area, while applying the iron nanoparticles coated on amorphous carbon (Fe-AC) to evaluate microwave absorption performance and discussed the relationship between structure and absorption property. The as-obtained ferrite carbon nanomaterials have some advantages as below: (I) a composite micron absorbent material with magnetic loss of ferrite and dielectric loss of amorphous carbon is designed; (II) the controllable structure possesses a well-proportioned micro-nanostructure; meanwhile, metal nanoparticles are well-dispersed on the surface of amorphous carbon; (III) the lightweight carbon materials improve absorption performance and significantly decrease the use of high-density ferrite; (IV) the precursor of ager can not only strengthen the interaction between ferrite nanoparticles and amorphous carbon to promote the dispersity of ferrite particles, but also enhance the concentrate of defects on the surface of Fe-AC, thereby improving microwave absorption performance. It is expected that such ferrite magnetic nanomaterials can be applied to efficiently eliminate electromagnetic interference problems.

## 2. Experimental Section

### 2.1. Materials

Ferrous sulfate (FeSO_4_·7H_2_O, 98.0+%), nickel sulfate (NiSO_4_·6H_2_O, 98.0+%), and agar powder were purchased from Wako, Japan. All the reagents were used without further purification, and all the solutions were prepared using distilled (DI) water.

#### 2.1.1. Preparation of Amorphous Carbon (Sample AC)

The carbon-based material was synthesized by pyrolysis of agar-gel precursors as carbon source. Typically, 1 g agar powder was mixed into 100 mL DI water and dissolved under vigorous magnetic stirring at 80 °C; then, the transparent gel solution was cooled down to room temperature (RT). Finally, the solid-state agar-gel was calcined in a muffle furnace at 500 °C for 3 h with heating rate of 2 °C/min under Ar atmosphere. The as-obtained black powder was labeled as AC.

#### 2.1.2. Preparation of Iron (Fe) Nanoparticles Coated AC (Sample Fe-AC)

A total of 1 g agar powder and 8.04 g FeSO_4_·7H_2_O were mixed and dissolved in 100 mL DI water under vigorous magnetic stirring at 80 °C; then, the transparent gel solution was cooled down to RT. Finally, the solid-state gel was calcined in a muffle furnace at 500 °C for 3 h with heating rate of 2 °C/min under Ar atmosphere. The sample was denoted as Fe-AC.

#### 2.1.3. Preparation of Mixed Iron (Fe)/ Nickel (Ni) Nanoparticles Coated AC (Sample Fe/Ni-AC)

A solution of 1 g agar powder, 8.04 g FeSO_4_·7H_2_O, and 7.21 g NiSO_4_·6H_2_O were mixed and dissolved in 100 mL DI water under vigorous magnetic stirring at 80 °C; then, the transparent gel solution was cooled down to RT. Finally, the solid-state gel was calcined in a muffle furnace at 500 °C for 3 h with heating rate of 2 °C/min under Ar atmosphere. The sample was denoted as Fe/Ni-AC.

### 2.2. Characterization

The sample of AC, Fe-AC, and Fe/Ni-AC was characterized by various analyses as follows: scanning electron microscopy (SEM, Hitachi SU8010) at 15 kV; meanwhile, the surface elemental compositions were quantified by X-ray spectrometry (EDX) (Horiba EMAX); transmission electron microscopy (TEM) was performed by a JEM-2100 F TEM JEOL with the operating voltage of 200 kV; X-ray diffraction (XRD) was analyzed by Rigaku Smartlab with a Cu-Kα radiation (λ = 1.5418 Å) in a 2θ range of 10–90° and a scanning rate of 10 °/min; nitrogen adsorption–desorption isotherm was performed at 77.3 K on a Nova 4200 (Quantachrome Inc, FL, USA) after the sample was evacuated at 300 °C for 12 h; Fourier transfer infrared (FT-IR) spectra were recorded on a Jasco FT/IR-4200 infrared spectrophotometer; XPS spectrum was obtained by an XPS analysis system (VG Thermo ESCALab220i-XL, VG Scientific, East Grinstead, UK); magnetization properties were studied using a vibrating sample magnetometer 7404 (VSM), (LakeShore, Westerville, OH, USA).

### 2.3. Microwave Absorbing Properties Measurement

The microwave absorbing performance was measured by a vector network analyzer (VNA, E5071C, Agilent) using the coaxial measurements in frequency range of 1–18 GHz. The complex permittivity *ε_r_* (*ε_r_* = *ε*′ – *jε*″) and permeability *µ_r_* (*µ_r_* = *µ*′ − *jµ*″) were tested via pressing into toroidal shape (Φ_out_ = 7.00 mm; Φ_in_ = 3.00 mm) in the microwave absorbing properties measurement. Herein, the reflection loss (RL) is calculated based on the *ε_r_* and *µ_r_* by the following equation [37,38]:

Based on

(1) $$\mathrm{RL}=20\log\;\left|\frac{Z_{\mathrm{in}}/Z_{o}-1}{Z_{\mathrm{in}}/Z_{o}+1}\right|$$

(2) $$\frac{Z_{\mathrm{in}}}{Z_{o}}={\left(\frac{\mu_{r}}{\varepsilon_{r}}\right)}^{1/2}\tan h\left(j\frac{2\pi ft}{c}{\left(\mu_{r}\varepsilon_{r}\right)}^{1/2}\right)$$

where *Z_in_* is input impedance, *Z*_o_ is the impedance of the air, *t* is the absorbent thickness, *f* is the frequency of the incident electromagnetic wave, and *c* is the velocity of electromagnetic waves in free space [39].

## 3. Results and Discussion

### 3.1. Morphology and Crystalline Phase

**Figure 1:**
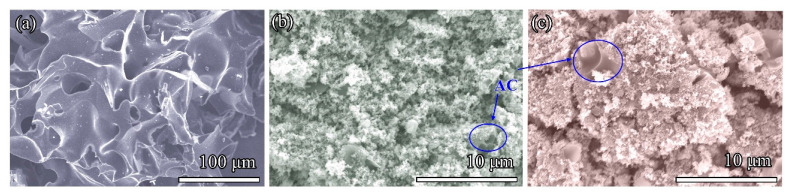
SEM images of the as-prepared samples: (**a**) AC, (**b**) Fe-AC, and (**c**) Fe/Ni-AC.

**Figure 2:**
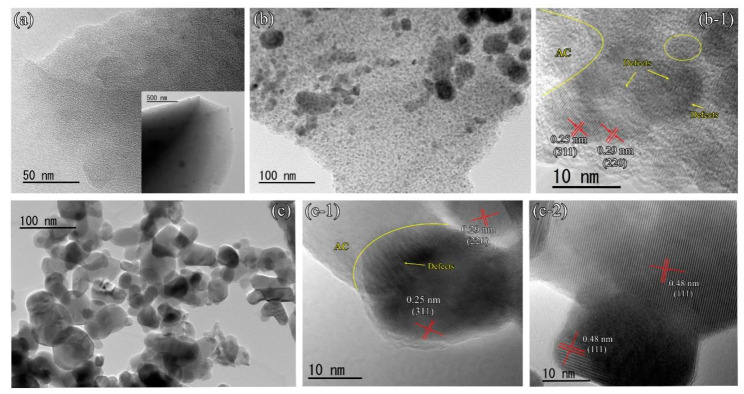
TEM and HRTEM images of (**a**) AC, (**b**) Fe-AC, and (**c**) Fe/Ni-AC. (Inset: the magnification of AC; (**b-1**,**c-1**,**c-2**) are different magnification areas in Figure 2b,c, respectively.)

The morphologies of as-prepared AC, Fe-AC, and Fe/Ni-AC samples were observed by SEM in Figure 1 and the elemental compositions were summarized in Table 1. It was interesting that the overall coral-like network structure was featured over the AC sample. However, the three-dimensional structures in Fe-AC and Fe/Ni-AC had random variable shapes along with the surface of AC. The similar morphologies of Fe-AC and Fe/Ni-AC were derived from the strong intermolecular forces between metal species and agar structures, which hindered the further assembly of crystal; meanwhile, more interface can be generated to increase the number of defects. More detailed geometrical structures of samples were further elucidated by TEM images. As shown in Figure 2a, the large layer structure of AC was observed in both low and high magnification. Furthermore, one can see that Fe-AC and Fe/Ni-AC were hierarchical structures assembled by particles in Figure 2b,c, respectively. The distinct lattice fringe of Fe-AC with an interplanar spacing of 0.25 and 0.29 nm in Figure 2b was in agreement with the spacing between the (311) and (220) planes of the Fe_2_O_3_ phase, respectively (JCPDS Card No. 39-1346). As shown in Figure 2c, the distinct lattice fringe of Fe/Ni-AC with an interplanar spacing of 0.25, 0.29, and 0.48 nm was in agreement with the spacing between the (311), (220), and (111) planes of the NiFe_2_O_4_, respectively (JCPDS Card No. 10-0325). Compared to Fe/Ni-AC, Fe-AC exhibited smaller particle sizes on the surface of AC, which was beneficial to the homogeneous distribution of Fe_2_O_3_ particles on the surface of carbon materials. Furthermore, as shown in Table 1, the specific surface areas of AC, Fe-AC, and Fe/Ni-AC were 20.71, 79, and 56.24 m^2^/g, respectively. With the doping of Ni^2+^, the BET of the Fe/Ni-AC decreased, which may be ascribed to the formation of a bigger particle size of NiFe_2_O_4_.

**Figure 3:**
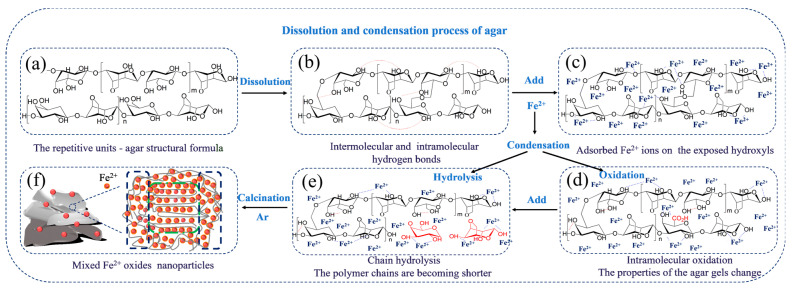
Schematic diagram of the condensation as agar soft template. (**a**) the agar structure before dissolution (**b**) the agar structure after dissolution into water (**c**) the agar structure after adding the adsorbed Fe^2+^ (**d**) the oxidation process of agar structure (**e**) the hydrolysis process of agar structure (**f**) the schematic diagram of Fe oxides and the atom sort order of Fe.

Notably, the abundant defects were traced by employing TEM analysis using agar as a soft template to provide the source of carbon. Especially, as shown in Figure 2(b-1,c-1,c-2), the defects were observed by discontinuous lattice fringes at the original orientation, which was caused by the chemical characters of agar. As shown in Figure 3, the agar including the repetitive units of D-galactose, 3,6-anhydrous-L-galactose were dissolved into water. Firstly, the units were aggregated by hydrogen bonds or Vander Waals forces [40]. Subsequently, the formation of ionic bonds became the meaningful intermolecular interactions between metal ions and polymer chains after Fe^2+^ and/or Ni^2+^ participation. As such, the relationship between metal species and carbon was established due to the strong interaction. In the process of agar-gel aging, agar gelation occurred in the agarose content, which is produced via hydrogen bonds; meanwhile, the structural change in repetitive units is characterized by a hydrolysis and oxidation reaction. The polymer chains of the agar chain are becoming shorter and shorter due to hydrolysis. The separation of small molecules may cause the abundant defects after calcination, which was beneficial to the absorption properties. For intermolecular oxidation in the aging process, oxygen was regarded as the crucial factor, which was responsible for the change in the functional group from hydroxyl groups, connected to primary carbon atoms, to carboxyl functions in the agar chain. These additional active defects, derived from the edge dislocation and mismatching of the agar structure, contributed more to the absorption performance than those of perfect crystallinity or polycrystalline materials. Herein, the carbonization of agar units supported the framework of Fe-AC and Fe/Ni-AC samples under Ar calcination at 500 °C. However, the Fe/Ni-AC had a bigger particle size and lower number of defects compared to that of the Fe-AC, which confirmed that the substitution of Ni^2+^ species into the mixed Fe oxide and carbon increased the crystallinity and decreased the concentration of defects.

**Figure 4:**
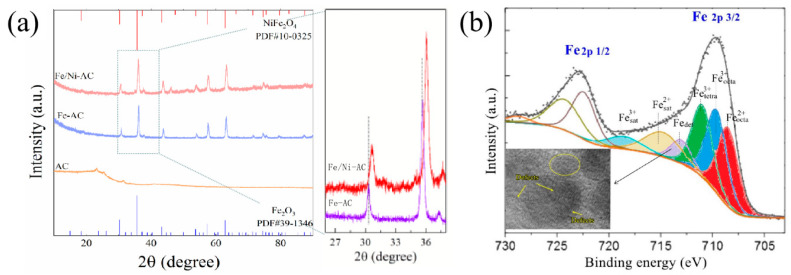
(**a**) XRD patterns of as-prepared AC, Fe-AC, and Fe/Ni-AC (inset: the enlarged parts in the 2θ range from 26° to 38°); (**b**) Fe 2p spectra profiles of Fe-AC.

The identity and crystal phase purity of AC, Fe-AC, and Fe/Ni-AC were detected by XRD, and the related results are shown in Figure 4a. In the range of 2θ at 20–30° and 40–45°, the broad peak was detected in AC, which belongs to the amorphous carbon, revealing the agar structure can be completely transformed to carbon in the annealing process at 500 °C for 3 h. For the Fe-AC sample, all diffraction peaks were indexed to the Fe_2_O_3_ (JCPDS 39-1346). There were no distinct shifts on the peaks at 2θ = 30.2°, 35.6°, 43.3°, 57.3°, and 62.9°, corresponding to the (220), (311), (400), (511), and (440) planes, respectively. After doping Ni^2+^ species into Fe-AC, the characteristic peaks of Fe/Ni-AC were similar to that of NiFe_2_O_4_ (JCPDS 10-0325) [41]. One can see that all prominent diffraction peaks shifted toward higher diffraction angles and the intensities became more substantial with increasing Ni-incorporating content, which may be due to the Ni species being entered into the Fe_2_O_3_ lattice and provoking the contraction of its unit cell. The results indicated that the existence of Ni could cause higher crystallinity of the mixed Fe-Ni particles. Moreover, as shown in Figure 4a, the characteristic peak of amorphous carbon disappeared after doping of Fe and Ni, due to the particles being able to cover the surface of AC.

Moreover, the Fe 2p spectrum was divided into eight peaks belonging to two regions at ca. 710 eV and 723 eV, respectively. Herein, the results of XPS focused on the Fe 2p_3/2_ region due to the low intensity and unstable errors of the Fe 2p_1/2_ region. The best sample (Fe-AC) was indexed to the Fe_2_O_3_, which consisted of Fe in two oxidation states (Fe^2+^ and Fe^3+^) and in two crystallographic structures (octahedral site and tetrahedral site). As shown in Figure 4b, three peaks can be ascribed to Fe^2+^_octa_, Fe^3+^_octa_, and Fe^3+^_tetra_, respectively. Especially, the peak of Fe_def_ was presented in the region of Fe 2p_3/2_, which connected with the defect structure in TEM images. It has an impact on the Fe state.

**Figure 5:**
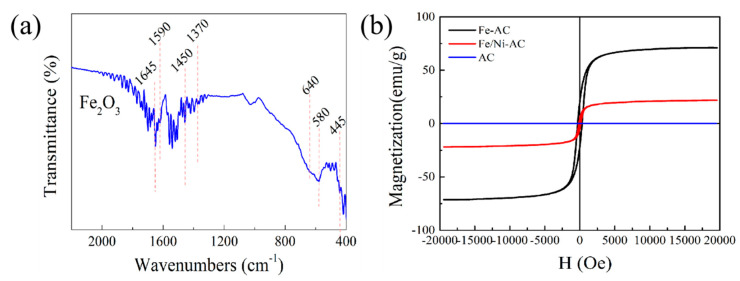
(**a**) FT-IR spectra of the synthesized Fe-AC; (**b**) magnetic hysteresis loops of the Fe-AC, Fe/Ni-AC, and AC.

To further characterize the Fe-AC, FT-IR spectra of Fe_2_O_3_ particles were measured [42]. As shown in Figure 5a, the typical FT-IR spectrum of Fe_2_O_3_ was located at 580 cm^−1^ assigned to the Fe–O stretching in magnetite lattice. The shoulder peak at 640 cm^−1^ together with the small peak at 445 cm^−1^ are attributed to Fe–O vibration in the surface oxidized layer in the magnetite Fe_2_O_3_ particles.

Magnetic properties of the Fe_2_O_3_ nanoparticles decorated on amorphous carbon (AC) nanocomposites were revealed by the hysteresis loop in Figure 5b. The saturation magnetizations of the Fe-AC, Fe/Ni-AC, and AC were 69.96 emu/g, 21.41 emu/g, and 0.00834 emu/g, respectively. It was obvious that the value of saturation magnetization decreased with the Ni addition amount, illustrating the negative role of Ni existence in the generation of Fe-AC for the achieving of good microwave absorption properties. Notably, Fe-AC possessed the strongest magnetization response in Figure 5b, which predicted the microwave absorption properties well, as shown in Section 3.3.

**Figure 6:**
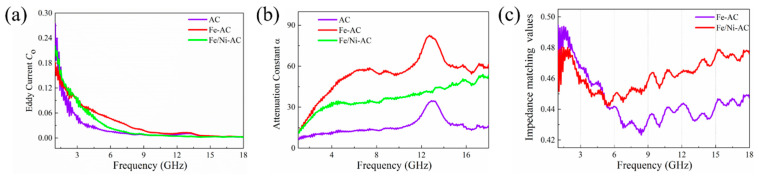
(**a**) Eddy current of AC, Fe-AC, and Fe/Ni-AC; (**b**) attenuation constants of AC, Fe-AC, and Fe/Ni-AC; (**c**) impedance matching coefficient of Fe-AC and Fe/Ni-AC.

As shown Figure 6a, the value of eddy current, evaluated by the equation $C_{o}=\mu^{\prime \prime }{\left(\mu^{\prime }\right)}^{-2}f^{-1}$ [43], decreased with violent fluctuations under high-frequency microwave band (1–18 GHz), which indicated there was no noteworthy contribution to electromagnetic wave absorption. In Figure 6b, the evaluation of the attenuation constant ″α″ is calculated by the equation $\frac{\sqrt{2}\pi f}{c}\sqrt{\left(\mu^{\prime \prime }\varepsilon^{\prime \prime }-\mu^{\prime }\varepsilon^{\prime }\right)+\sqrt{{\left(\mu^{\prime \prime }\varepsilon^{\prime \prime }-\mu^{\prime }\varepsilon^{\prime }\right)}^{2}+{\left(\mu^{\prime }\varepsilon^{\prime \prime }+\mu^{\prime \prime }\varepsilon^{\prime }\right)}^{2}}}$ [44]. The value ″α″ of the as-prepared Fe-AC and Fe/AC-Ni nanocomposites was higher than AC nanocomposite, meaning that the Fe-AC and Fe/AC-Ni nanocomposites have a better shielding property. It can be seen in Figure 6c that Fe/Ni-AC provided a high impedance matching coefficient which can be evaluated by using the following equation $\eta =\left|\frac{Z_{\mathrm{in}}}{Z_{o}}\right|$ [45]. Herein, the doping of Ni could effectively improve impedance matching and reduce surface depth, thereby controlling the microwave absorption of the materials.

### 3.2. Complex Permittivity (ε_r_) and Permeability (u_r_)

The imaginary parts (*ε*″and *μ*″) and real parts (*ε*′ and *μ*′) of the complex permittivity *ε_r_* and permeability *u_r_* symbolize the loss and storage of magnetic energy and electrical energy, respectively [46].

**Figure 7:**
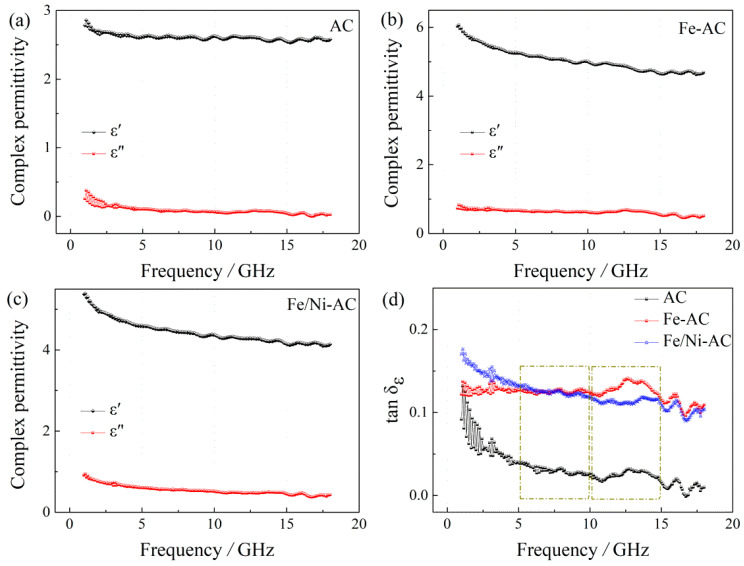
Complex permittivity (*ε_r_*) of real parts (*ε*′) and imaginary parts (*ε*″) of: (**a**) AC, (**b**) Fe-AC, and (**c**) Fe/Ni-AC, and (**d**) their tan *δ_ε_* = *ε*″/*ε*′.

As shown in Figure 7, the complex permittivity of AC, Fe-AC, and Fe/Ni-AC is obtained in the frequency range of 1–18 GHz. We can see that the three samples exhibit complex permittivity at different levels and demonstrate other dielectric loss characteristics. In Figure 7a, the AC has the lowest complex permittivity, where *ε*′ drops from 2.86 at 1.0 GHz to 2.58 at 18.0 GHz and *ε*″ fluctuates slightly between 0.1 and 0.3 in the frequency range studied. With the adjustment of elemental species (Fe/Ni), both *ε*′ and *ε*″ are significantly enhanced. As an example, Figure 7b,c show the *ε*′ values of Fe-AC and Fe/Ni-AC decrease from 6.13 to 4.61 and 5.41 to 4.07, respectively; the *ε*″ values of Fe-AC and Fe/Ni-AC decline from 0.87 to 0.43 and 0.96 to 0.35, respectively. In Figure 7d, it can be found that dielectric loss of Fe-AC and Fe/Ni-AC can change in a reasonably limited range by modulation of the dielectric loss tangents (tan *δ_ε_* = *ε*″/*ε*′). Finally, it is also found that the tan *δ_ε_* have two resonances of the three samples in the range of 6–9 GHz and 11–14 GHz.

**Figure 8:**
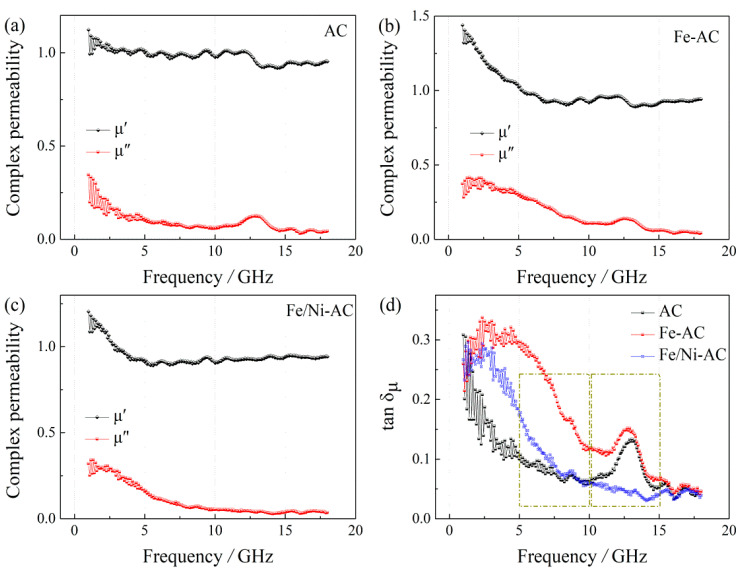
Complex permeability (*µ_r_*) of real parts (*µ′*) and imaginary parts (*µ*″) of: (**a**) AC, (**b**) Fe-AC, and (**c**) Fe/Ni-AC, and (**d**) their tan *δ_µ_* = *µ*″/*µ′*.

As shown in Figure 8a–c, although the *µ*′ values of the three samples are slightly different, their *µ*″ values are very close, which means that they have similar magnetic loss characteristics. In Figure 8d, this assumption that the three samples have similar magnetic loss characteristics and can be found to be supported by the magnetic loss tangents (tan *δ_µ_* = *µ*″/*µ*′), varies over a relatively limited range. Hence, it is also found that the tan$\;\delta_{\mu }$ has two resonances of the three samples in the range of 6–9 GHz and 11–14 GHz.

### 3.3. Microwave Absorption Properties

Microwave absorption properties of three samples are highly relevant to its complex permittivity and complex permeability; therefore, based on the measurement data of the complex permittivity and complex permeability (Figure 7 and Figure 8), you can evaluate RL properties. In the above Formulas (1) and (2) of Section 2.3, it can be found that the RL properties depend on the absorbent thickness *t*, so further investigation of the different thicknesses (3.0 mm, 3.5 mm, 4.0 mm, 4.5 mm, 5.0 mm, and 5.5 mm) results in reflective losses to eliminate the effects of absorber thickness.

**Figure 9:**
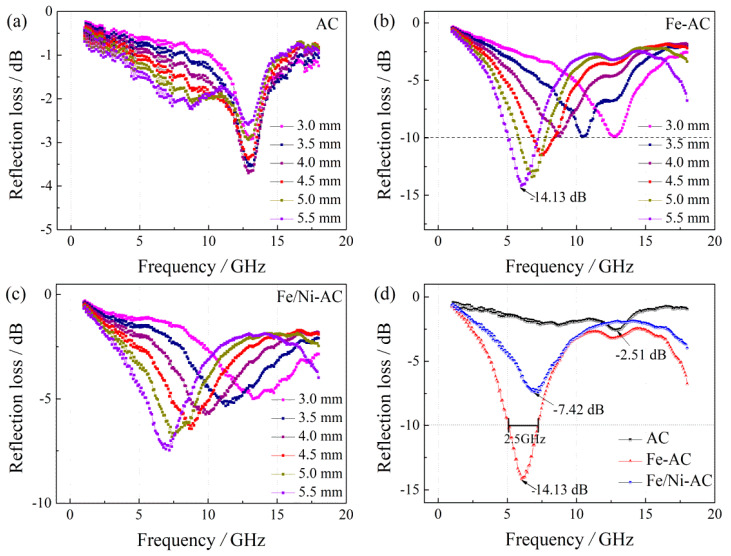
Reflection losses of (**a**) AC, (**b**) Fe-AC, and (**c**) Fe/Ni-AC (*t* from 3.0 mm to 5.5 mm), and (**d**) the reflection losses (*t* = 5.5 mm) of those samples.

As shown in Figure 9a–c, Fe-AC not only maintains a wider bandwidth width of −10 dB over AC and Fe/Ni-AC at various absorbent thicknesses *t*, but also maintains the advantage of RL maximums as the thickness increases. Fe-AC also exhibits negative shifts to low frequency as the thickness increases, but the maximum value of its RL properties is less affected by the thickness change. Therefore, Fe-AC can be the best choice as a good microwave absorbent in this work. This considered, the integrated absorbent thickness produces a fairly wide bandwidth in the 5–13 GHz range for Fe-AC. Figure 9d shows the calculated RL properties of Fe-AC with a thickness of 5.5 mm in the frequency range of 1–18 GHz. The maximum RL value for Fe-AC was −14.13 dB at 6.0 GHz and the bandwidth over −10 dB (90% absorption [47]) was 2.5 GHz.

## 4. Conclusions

In this work, the ferrite magnetic nanomaterials with rich defect structures were prepared for the elimination of electromagnetic interference via a facile agar-gel strategy, which can be applied to ensure the communication between deep-diving research vehicles. It was found that the element contents of Fe and Ni species greatly affect sample properties, such as particle size, morphology, crystalline phase, and absorbing performance of ferrite magnetic nanomaterials. Among them, the Fe-AC exhibited the highest RL performance with −14.13 dB at 6.0 GHz and bandwidths over −10 dB (90% absorption) for them are 2.5 GHz. According to the characterizations of the Fe-AC nanoparticles, it was concluded that the improved absorbing performance was due to these more additional active defects, smaller nanoparticle distribution, and higher surface area than that of AC and Fe/ Ni-AC. As expected, a facile soft-template agar-gel strategy could be used for the designing of novel microwave absorption materials in the near future.

## Figures and Tables

**Table 1:** Elemental compositions on the surfaces based on EDX analysis. BET surface areas for AC, Fe-AC, and Fe/Ni-AC.

Sample	EDX Element Mapping (wt. %)			BET (m²/g)	D (nm)	V (cm^3^/g)
	Fe	Ni	O			
AC	/	/	/	20.71	3.78	0.013
Fe-AC	70.2	/	29.8	79	3.41	0.13
Fe/Ni-AC	42.2	35.6	22.2	56.24	3.83	0.15

## Data Availability

Not applicable.
